# Upper Limb Ataxia Among Residents With Chronic Inorganic Arsenic Exposure: A Quantitative Pilot Study

**DOI:** 10.7759/cureus.100304

**Published:** 2025-12-29

**Authors:** Yuma Sato, Nobuyuki Ishii, Takashi Sugiyama, Kazutaka Shiomi, Taiga Miyazaki, Hitoshi Mochizuki

**Affiliations:** 1 Department of Neurology, Faculty of Medicine, University of Miyazaki, Miyazaki, JPN; 2 Department of Neurology, Chiyoda Hospital, Hyuga, JPN; 3 Department of Internal Medicine, Division of Respirology, Rheumatology, Infectious Diseases, and Neurology, Faculty of Medicine, University of Miyazaki, Miyazaki, JPN; 4 Department of Neurology, Ebihara General Hospital, Takanabe, JPN

**Keywords:** cerebellar ataxia, digital biomarkers, dizziness, elderly population, inorganic arsenic exposure, peripheral neuropathy, smartphone spiral analysis, toroku japan

## Abstract

Background

Organic arsenic compounds can cause cerebellar ataxia, but the neurological profile of chronic inorganic arsenic exposure remains unclear. In Toroku, Japan, a historically exposed community, many residents report dizziness, yet it is unknown whether chronic inorganic arsenic exposure is associated with cerebellar dysfunction. We focused on upper limb ataxia as an accessible and quantifiable manifestation of cerebellar function in this setting.

Methods

We conducted a retrospective pilot study of residents who had lived within 1,000 meters of the Toroku mine roaster before 1962 and were chronically exposed to airborne inorganic arsenic. All participants belonged to this exposed cohort; no unexposed control group was included. Participants aged 66-85 years who could perform a spiral-drawing task were classified as certified with chronic arsenic poisoning (C-CAP) or non-certified (NC) participants, based on Japanese government criteria. Upper limb coordination was quantified using the validated smartphone application TREMOR AI (Densan Software, Co., Ltd., Miyazaki, Japan), which computes deviation area (mm²) and percentage spiral length (%). An experienced neurologist, blinded to TREMOR AI results, assessed clinical cerebellar ataxia and peripheral neuropathy. Group comparisons used the Mann-Whitney U test, Fisher’s exact test, and rank biserial correlations.

Results

Thirty-two residents were included (17 C-CAP and 15 NC). C-CAP participants tended to be older than NC participants, but demographics and metabolic factors were otherwise comparable. Clinically evident cerebellar ataxia was rare in both groups, whereas distal symmetric peripheral neuropathy was common (64.7% vs 60.0%). No significant between-group differences were observed in % spiral length (p = 0.484, r = 0.127) or deviation area (p = 0.970, r = 0.010), indicating no large differences in upper limb ataxia between certified and non-certified exposed residents.

Conclusions

Within this chronically exposed Japanese cohort, chronic inorganic arsenic poisoning was not primarily characterized by prominent upper limb ataxia on either clinical examination or smartphone-based spiral analysis. The combination of frequent distal symmetric neuropathy and age-related vestibular decline may contribute to dizziness in some residents, supporting a multisensory deficit hypothesis that warrants testing in future vestibular and postural studies.

## Introduction

Chronic inorganic arsenic exposure from contaminated groundwater is a global health threat affecting 94-220 million people worldwide [1,2]. While organic arsenic is a known cause of cerebellar ataxia, a severe neurological complication [1], the specific neurological effects of chronic inorganic arsenic exposure remain poorly understood. This knowledge gap leaves a fundamental clinical question unanswered: whether chronic inorganic arsenic exposure leads to cerebellar dysfunction comparable to that seen with organic arsenic. This uncertainty presents a significant challenge in developing regions with limited access to neurological specialists.

To address this question, we studied a unique cohort in Toroku, Japan, a historic site of inorganic arsenic poisoning officially recognized as a "Kogai" (pollution-related disease) [3]. Our university has provided long-term neurological care to these residents since 1975, offering a well-characterized population for investigation [1,4,5]. Clinically, residents frequently report dizziness, yet its underlying pathophysiology is unknown, and it has not been determined whether this exposure causes cerebellar dysfunction [5]. In this context, upper limb ataxia represents one accessible and quantifiable manifestation of cerebellar dysfunction that can be specifically targeted for objective assessment.

Investigating subtle motor deficits is challenging because conventional neurological examinations can be subjective. Several clinical rating scales, such as the International Cooperative Ataxia Rating Scale (ICARS) and the Scale for the Assessment and Rating of Ataxia (SARA), have been developed to standardize the assessment of cerebellar ataxia [6,7]. To overcome this challenge in our setting, we employed TREMOR AI (Densan Software, Co., Ltd., Miyazaki, Japan), a validated, smartphone-based application that quantifies spiral-drawing performance as an objective measure of motor coordination [8]. The application's deviation score correlates significantly with the ICARS, making it a practical and accessible tool, especially in resource-limited settings.

Therefore, this pilot study used this objective tool to quantitatively investigate upper limb ataxia within a cohort of residents chronically exposed to inorganic arsenic and to compare certified patients with chronic arsenic poisoning (C-CAP) with similarly exposed but non-certified residents (NC). Because individual biomonitoring data were not available, we used residential proximity and administrative certification as pragmatic proxies for long-term exposure and designed the study as a within-cohort comparison. We focused on upper limb coordination as one component of cerebellar function and aimed to determine whether certified patients show additional subtle motor deficits beyond those observed in other exposed residents, as a first step toward clarifying the neurological basis of symptoms reported by these residents, such as dizziness.

## Materials and methods

This retrospective cohort study was approved by the University of Miyazaki Ethics Committee, which waived informed consent. The study adhered to the Declaration of Helsinki principles. Participant evaluations and data collection for this retrospective cohort were conducted between November 8, 2022 and December 31, 2023. The reporting of this observational study follows the STROBE (Strengthening the Reporting of Observational Studies in Epidemiology) guidelines [9]. Because arsenic exposure was widespread among residents of Toroku, the study did not include an unexposed control group; instead, it focused on differences between C-CAP and similarly exposed NC residents.

We enrolled residents of Toroku, Japan, who had lived within 1,000 meters of the mine roaster before 1962. Participants were grouped as certified (C-CAP) or non-certified (NC) for chronic arsenic poisoning based on official government criteria, which require specific skin, nasal, or neurological signs in conjunction with exposure history [3]. In brief, certification is determined according to governmental guidance based on documented exposure history plus a defined combination of characteristic arsenic-related clinical manifestations (e.g., dermatologic and/or nasal lesions) together with neurological findings (most commonly distal symmetric neuropathy), as detailed in the official criteria [3]. The C-CAP group comprised residents who fulfilled these criteria and were officially recognized as patients with pollution-related disease. The NC group consisted of chronically exposed residents who had undergone clinical evaluation because of their exposure or health concerns but did not meet the full combination of findings required for certification; some of them had mild symptoms such as sensory complaints or dizziness, whereas others were minimally symptomatic. Inclusion criteria for this analysis were an age of 66-85 years and the ability to perform the spiral-drawing task. No additional systematic exclusion criteria (e.g., alcohol use) were applied beyond the inability to complete the task.

Environmental arsenic exposure in Toroku has been described in detail elsewhere [3-5]. Briefly, residents were chronically exposed to airborne inorganic arsenic emitted from the mine roaster until its closure in 1962. In the present study, we did not have individual quantitative indices of cumulative exposure, such as measured arsenic concentrations or precise exposure duration. Instead, residence within 1,000 meters of the roaster before 1962, together with the administrative certification status, was used as a proxy for long-term inorganic arsenic exposure.

As potential clinical covariates, we also obtained a history of regular drinking from medical records, defined as alcohol consumption on multiple days per week, and laboratory markers of glucose metabolism (blood sugar and HbA1c) at the time of evaluation, given their relevance to peripheral neuropathy and motor function. "Regular drinking" was recorded as a binary variable based on documentation in the medical record; detailed quantity (e.g., grams/day) was not consistently available in this retrospective dataset. Participants were not excluded based on alcohol use or metabolic factors; these variables were collected to support clinical interpretation as potential confounders.

Upper limb motor coordination was assessed using our validated TREMOR AI smartphone application. Participants traced a predefined spiral three times. The median deviation area (mm²) and % spiral length (%) from the three trials were used for analysis. The TREMOR AI deviation-based metrics have been previously validated in clinical cohorts and have been shown to correlate with the ICARS [8].

All assessment tools used in this study were available for non-commercial academic use without additional licensing fees. The clinical ataxia rating scales (ICARS and SARA) are established research tools that do not require a specific license for observational studies [6,7], and the TREMOR AI application was developed within our institution and used under an internal, non-commercial research agreement [8]. No proprietary commercial scoring systems were employed.

Additionally, all participants underwent a standardized neurological examination by an experienced neurologist blinded to the TREMOR AI results. Based on this examination, the presence or absence of two key clinical findings was recorded: (i) cerebellar ataxia, defined by clear dysmetria or intention tremor, and (ii) peripheral neuropathy, defined by symmetric distal sensory deficits and/or reduced or absent ankle tendon reflexes on clinical examination. Nerve conduction studies were not performed systematically, and the clinical definition did not distinguish between arsenic-related and other potential etiologies of neuropathy. The presence of self-reported dizziness was also ascertained. Dizziness was recorded as a binary self-report item, and detailed information on severity, duration, or provoking factors was not systematically collected.

Statistical analyses used R version 4.3.3 (R Foundation for Statistical Computing, Vienna, Austria, https://www.R-project.org/). Groups were compared using the Mann-Whitney U test for continuous variables and Fisher's exact test for categorical variables. The rank biserial correlation (r) was calculated as an effect size for the Mann-Whitney U test. A two-sided P-value < 0.05 was considered statistically significant. Given the exploratory nature and limited sample size of this pilot study, no formal a priori sample size calculation or equivalence margin was specified, and effect sizes were emphasized to aid interpretation of non-significant findings.

## Results

Baseline characteristics of the 32 participants (17 C-CAP, 15 NC) are shown in Table 1. The groups were comparable in demographics and clinical history, though a trend towards an age difference was observed (p = 0.069, r = 0.325). The prevalence of regular drinking was similar between groups (35.3% in C-CAP vs. 46.7% in NC), and blood sugar and HbA1c levels did not differ significantly, suggesting no major imbalances in alcohol use or glucose metabolism that might confound group comparisons.

**Table 1:** Baseline characteristics and clinical findings of participants. Data are presented as median (IQR) for continuous variables, and n (%) for categorical variables. Mann–Whitney U test was used for continuous variables; Fisher’s exact test for categorical variables. Regular drinking was defined as alcohol consumption on multiple days per week. IQR: interquartile range

Parameters	Certified Participants (C-CAP) (n = 17)	Non-certified Participants (NC) (n = 15)	Statistical Comparison
Demographics			
Age (years), median (IQR)	78 (74-81)	73 (68-79)	Mann-Whitney U = 79.0; p = 0.069
Sex (male), n (%)	10 (58.8%)	5 (33.3)	Fisher’s exact; p = 0.178
History of regular drinking, n (%)	6 (35.3%)	7 (46.7)	Fisher’s exact; p = 0.720
Clinical Findings			
Peripheral neuropathy, n (%)	11 (64.7%)	9 (60.0)	Fisher’s exact; p > 0.999
Cerebellar ataxia (clinical), n (%)	0 (0%)	1 (6.7)	Fisher’s exact; p = 0.469
Dizziness, n (%)	3 (17.6%)	4 (26.7)	Fisher’s exact; p = 0.678
Laboratory Data			
Blood sugar (mg/dl), median (IQR)	100 (98-116)	99 (90-103)	Mann-Whitney U = 93.0; p = 0.198
HbA1c (%), median (IQR)	5.8 (5.5-6.0)	5.6 (5.2-6.1)	Mann-Whitney U = 106.0; p = 0.427

Clinically, a high prevalence of peripheral neuropathy was observed in both groups (64.7% in C-CAP, 60.0% in NC) based on the predefined clinical criteria, with no significant difference. In contrast, clinically evident cerebellar ataxia was rare and did not differ between the groups (Table 1). Thus, the NC group in this study should not be regarded as a completely asymptomatic exposed population but rather as residents with varying degrees of clinical involvement who did not satisfy the strict administrative criteria for certification.

**Figure 1:**
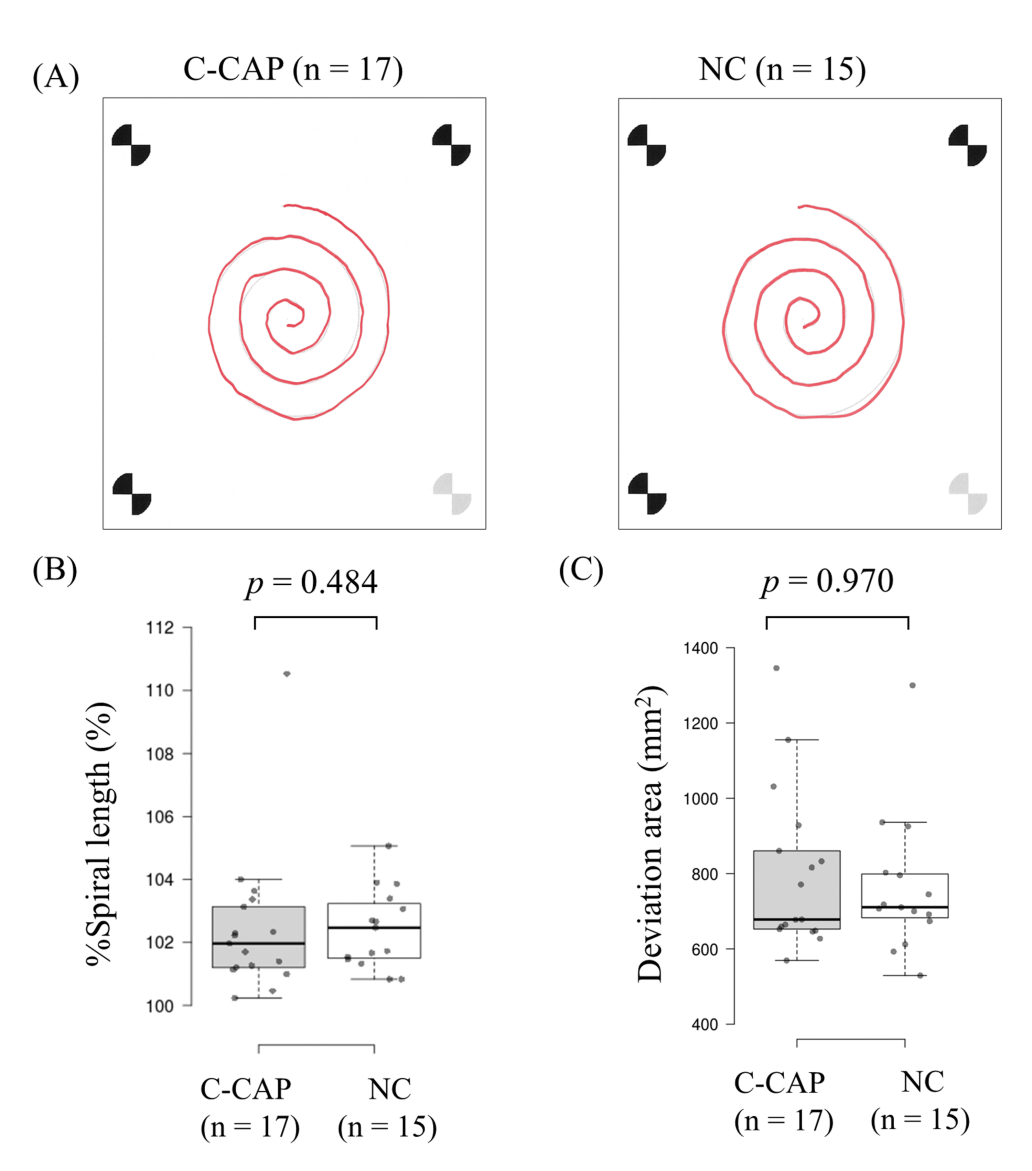
Quantitative assessment of upper limb motor coordination using TREMOR AI. (A) Representative spiral drawings from a certified participant with chronic arsenic poisoning (C-CAP) and a non-certified participant (NC). These examples were chosen because their deviation area scores were close to the median value of their respective groups. TREMOR AI (Densan Software, Co., Ltd., Miyazaki, Japan) is a validated smartphone-based application for quantitative analysis of spiral drawing performance [8]. (B, C) Box plots comparing the % spiral length and deviation area between the C-CAP (n = 17) and NC (n = 15) groups. The box plots display the median (center line), interquartile range (box limits), and minimum/maximum values (whiskers). Individual data points for each participant are superimposed as dots. P values were calculated using the Mann–Whitney U test.

The quantitative TREMOR AI results (Figure 1) were consistent with these clinical findings. No significant between-group differences were found for either % spiral length (p = 0.484) or deviation area (p = 0.970). The corresponding effect sizes for these comparisons were negligible (r = 0.127 and r = 0.010, respectively), indicating that any potential differences in upper limb ataxia between certified and non-certified exposed residents are unlikely to be large in magnitude within this sample.

## Discussion

The central finding of this pilot study is that, within a cohort of residents chronically exposed to inorganic arsenic, C-CAP participants did not exhibit greater upper limb ataxia than similarly exposed but non-certified residents. Because individual biomonitoring data were not available and neurological findings in older adults may have mixed etiologies, our data do not establish arsenic-specific causality; rather, they support a pragmatic within-cohort comparison focused on upper limb coordination.

Our quantitative and clinical assessments revealed no significant differences in ataxic signs between the C-CAP and NC groups, and no evidence of marked upper limb ataxia in either group. Notably, the C-CAP group tended to be older than the NC group, and aging is generally associated with worse performance on fine motor and drawing tasks. Despite this age-related disadvantage, C-CAP participants did not show poorer spiral-drawing performance or more frequent upper limb ataxia than NC participants, which supports the notion that certification status, reflecting greater clinical severity of chronic inorganic arsenic poisoning, is not associated with additional upper limb cerebellar dysfunction within this exposed cohort. This finding contrasts with the neurological profile of other heavy metal toxicities and poisoning from organic arsenic [10,11], suggesting a potentially different neurological profile with respect to appendicular ataxia in this setting. However, because our assessment focused on upper limb coordination, these findings do not exclude the possibility of cerebellar involvement in other domains, such as gait or truncal stability. In addition, because all participants in this study were chronically exposed residents and we did not include an unexposed age-matched control group, we cannot exclude the possibility that subtle, symmetric upper limb ataxia may be present to a similar extent in both certified and non-certified residents.

This apparent absence of appendicular ataxia is broadly compatible with available toxicological evidence. Preclinical studies comparing organic arsenicals, such as diphenylarsinic acid, with inorganic arsenic compounds have reported greater accumulation of the organic species in brain tissue and more pronounced cerebellar toxicity, whereas inorganic arsenic tends to show predominantly peripheral and systemic effects at comparable doses [12,13]. These findings suggest that the central nervous system, and particularly the cerebellum, may be relatively less exposed to inorganic arsenic than to certain organic arsenicals, although they do not exclude central effects at higher concentrations or in susceptible individuals. Our quantitative data are in line with this interpretation; the negligible effect size for deviation area (r = 0.010) does not support a large or clinically obvious effect of certification status on upper limb ataxia in this exposed cohort, although small differences cannot be excluded given the limited sample size.

In contrast to the absence of upper limb ataxia, peripheral neuropathy was highly prevalent in both groups (64.7% vs. 60.0%). This apparent paradox, in which a clinical finding used as part of the certification criteria is equally frequent in certified and non-certified residents, can be understood in light of the official guidelines [3]: certification requires the coexistence of neuropathy with arsenic-related skin manifestations and other features. These findings suggest that clinically defined distal symmetric neuropathy is common among residents with chronic inorganic arsenic exposure, regardless of certification status. However, given the advanced age of the cohort and the presence of metabolic risk factors such as altered glucose metabolism and regular alcohol use in some participants, it is likely that the observed neuropathy reflects a combination of arsenic-related and age- or metabolism-related processes rather than a neuropathy that is specific to arsenic alone [14,15].

In interpreting these findings, it is important to consider both exposure assessment and potential confounders. Individual cumulative arsenic exposure could not be quantified in this study, and residence within 1,000 meters of the roaster prior to 1962, plus certification status, was used as a proxy for long-term exposure. Furthermore, although we collected information on regular alcohol use and basic markers of glucose metabolism, other potential causes of neuropathy and ataxia, such as long-standing diabetes, nutritional deficiencies, or cerebrovascular disease, were not systematically assessed. Consequently, some cases of peripheral neuropathy or subtle motor impairment may reflect age-related or metabolic factors in addition to, or instead of, arsenic exposure.

The high prevalence of peripheral neuropathy in this cohort raises the possibility that dizziness reported by some residents may arise, at least in part, from a multisensory deficit rather than from primary cerebellar dysfunction. We therefore propose a multisensory dizziness hypothesis, in which impaired proprioceptive input from distal neuropathy could interact with age-related vestibular decline (presbyvestibulopathy) to compromise spatial orientation [16]. In the present study, however, vestibular function tests and quantitative assessments of dizziness severity or chronicity were not performed, and our data do not directly test this hypothesis. While the prevalence of dizziness in our sub-cohort was lower than previously reported, likely due to sampling bias [1,4,5], the multisensory framework remains a hypothesis-generating perspective for future studies and for clinical assessment of those patients who do experience dizziness. Within this context, our findings suggest that dizziness in chronically exposed residents should not be attributed automatically to cerebellar dysfunction and that a broader diagnostic workup, including evaluation of peripheral and vestibular function, may be useful.

This study has several limitations. These limitations define the scope of inference and indicate that our findings should be interpreted as evidence from a pragmatic within-cohort comparison rather than as causal or exposure-response conclusions. First, the design and exposure assessment were constrained. The sample size was small, the study was retrospective, and classification relied on administrative certification rather than direct exposure measures. We lacked individual-level data on cumulative arsenic dose, such as environmental concentrations or biological monitoring, and therefore used residential proximity to the roaster and certification status as surrogate indicators of long-term exposure. Because of the limited sample size, the study was underpowered to reliably detect small to moderate differences in upper limb ataxia between groups and was not designed as a formal equivalence trial; our findings should thus be interpreted as showing no evidence of large differences rather than proving the complete absence of any effect. In addition, arsenic exposure was widespread among Toroku residents, and no unexposed control group was included. As a result, we cannot determine whether upper limb ataxia is increased compared with the general population, and our conclusions are restricted to comparisons within the exposed cohort; mild symmetric upper limb ataxia affecting both groups to a similar degree also cannot be completely ruled out.

Second, there are important limitations in outcome characterization. Peripheral neuropathy was defined purely on clinical grounds without systematic nerve conduction studies, and we did not distinguish between arsenic-related and other etiologies such as long-standing diabetes, alcohol use, or age-related neuropathy. The high prevalence of neuropathy should therefore be interpreted as reflecting the overall burden of distal symmetric neuropathy in this exposed and elderly population rather than a neuropathy specific to arsenic. Assessment was also limited to upper limb coordination, and gait and truncal ataxia, which are other core features of cerebellar dysfunction, were not evaluated. Accordingly, a broader cerebellar syndrome affecting balance and walking cannot be definitively excluded, and our conclusions should be interpreted as applying specifically to upper limb coordination rather than cerebellar function as a whole. Moreover, vestibular function and postural control were not systematically assessed, so our proposed multisensory dizziness hypothesis remains speculative and hypothesis-generating rather than a conclusion directly supported by the present data.

Finally, generalizability and toxicological interpretation are limited. Our findings are derived from a single Japanese cohort, and it is uncertain whether they can be generalized to populations with different genetic backgrounds or environmental co-exposures. In addition, our toxicological interpretation of relatively limited cerebellar involvement of inorganic arsenic is extrapolated from preclinical studies using specific arsenic species and exposure conditions, because direct measurements of central nervous system arsenic levels were not available in this cohort.

## Conclusions

In this quantitative pilot study of a Japanese cohort chronically exposed to inorganic arsenic, chronic inorganic arsenic poisoning was not primarily characterized by prominent upper limb ataxia on either clinical examination or smartphone-based spiral analysis. The combination of frequent distal symmetric neuropathy and age-related vestibular decline may contribute to dizziness in some residents, and our findings are consistent with, but do not prove, a multisensory deficit mechanism that remains to be tested in dedicated vestibular and postural studies. Taken together, these observations support a more multifactorial approach to the neurological assessment of chronically arsenic-exposed populations and highlight the need to look beyond cerebellar dysfunction alone when evaluating dizziness in this context.
